# Effects of cervical rotatory manipulation on the cervical spinal cord: a finite element study

**DOI:** 10.1186/s13018-021-02885-6

**Published:** 2021-12-24

**Authors:** Fan Xue, Zujiang Chen, Han Yang, Taijun Chen, Yikai Li

**Affiliations:** 1 School of Traditional Chinese Medicine, Southern Medical University, Baiyun District, Guangzhou, Guangdong Province China; 2 Zunyi Medical and Pharmaceutical College, Pingan District, Zunyi, Guizhou Province China

**Keywords:** Cervical rotatory manipulation, Spinal cord, Finite element analysis, Biomechanics

## Abstract

**Background:**

Little information is available concerning the biomechanism involved in the spinal cord injury after cervical rotatory manipulation (CRM). The primary purpose of this study was to explore the biomechanical and kinematic effects of CRM on a healthy spinal cord.

**Methods:**

A finite element (FE) model of the basilaris cranii, C1–C7 vertebral bodies, nerve root complex and vertebral canal contents was constructed and validated against in vivo and in vitro published data. The FE model simulated CRM in the flexion, extension and neutral positions. The stress distribution, forma and relative position of the spinal cord were observed.

**Results:**

Lower von Mises stress was observed on the spinal cord after CRM in the flexion position. The spinal cord in CRM in the flexion and neutral positions had a lower sagittal diameter and cross-sectional area. In addition, the spinal cord was anteriorly positioned after CRM in the flexion position, while the spinal cord was posteriorly positioned after CRM in the extension and neutral positions.

**Conclusion:**

CRM in the flexion position is less likely to injure the spinal cord, but caution is warranted when posterior vertebral osteophytes or disc herniations exist.

## Background

Cervical spine manipulation (CSM) therapy is considered an effective treatment for musculoskeletal ailments such as nonspecific mechanical neck pain [1, 2]. In recent years, the clinical efficacy and possible mechanisms of CSM have been increasingly studied [3]. Cerebrovascular accidents, spinal cord injury and nerve root injury are the three most common side effects or complications following CSM [4, 5]. However, little information is available concerning the biomechanism involved in the side effects or complications of CSM.

Cervical rotatory manipulation (CRM), a widely used CSM technique in China developed by Feng [6], is comparable to the high-velocity thrust cervical techniques of Western medicine [7]. Our previous studies showed that CRM can affect the nucleus pulposus, carotid atherosclerotic plaques and vascular haemodynamic properties [8–10]. Spinal cord injury is also a serious accident in CRM [5]. However, the stress, strain and relative displacement of the healthy spinal cord during CRM have not been explored. In China, CRM in the flexion position is widely used, as the flexion position is considered to be a safer position than the extension or neutral positions during this procedure. However, whether CRM in the flexion position is suitable for all patient conditions has not been explored. Accordingly, the objective of this study was (1) to explore the biomechanical and kinematic effects of CRM on the spinal cord in the flexion, extension and neutral positions and (2) to explore the relationship between the effects and the clinical applications of CRM. The finite element (FE) analysis method was used in this study.

## Methods

### Model construction

We enrolled a healthy male volunteer (32 years old, 175 cm and 68 kg) without craniocervical diseases (cervical disc herniation, fracture, tumour, etc.) based on clinical symptoms, physical examination and X-ray examination. A 3-dimensional (3D) craniocervical FE model, including the basilaris cranii, C1–C7 vertebral bodies, intervertebral disc, zygapophysial cartilage, nerve root complex and vertebral canal contents (spinal cord, pia matter, dura matter, cerebrospinal fluid (CSF) and denticulate ligaments (DLs)), was reconstructed based on the computed tomography (CT) images (Dual Source 128, Siemens, Germany) of the healthy male volunteer at 0.625 mm intervals and published anatomical data of human vertebral canal contents [11, 12].

The basic geometry of basilaris cranii and C1–C7 vertebral bodies was reconstructed on the basis of the CT images obtained before using Mimics software 19.0 (Materialise, Leuven, Belgium). After that, an STL format file was imported into Geomagic Wrap 2017 software (Raindrop, Marble Hill, New York) to obtain a high-quality nonuniform rational B-splines (NURBS) surface model.

**Fig. 1:**
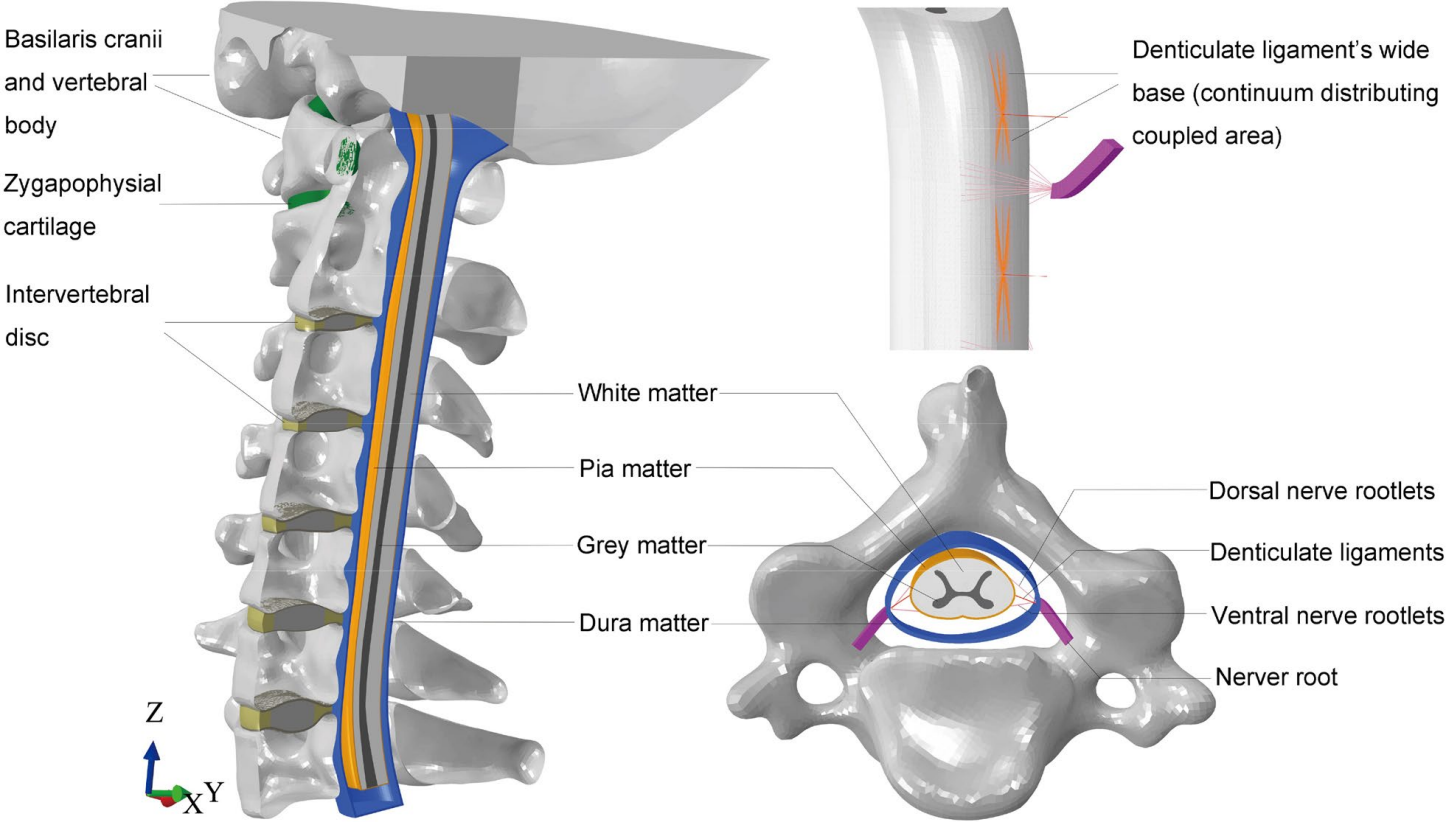
Intact FE model of the basilaris cranii, C1–C7 vertebral bodies, nerve root complex and vertebral canal contents

The geometries of other structures (intervertebral disc, zygapophysial cartilage, nerve root complex and vertebral canal contents), which were difficult to distinguish clearly from the grey value of CT images, were constructed using Solidworks 2017 software (Dassault Systems SA, Waltham, Massachusetts) based on published anatomical data [11, 12]. Specifically, the intervertebral disc was partitioned into the annulus fibrosus, nucleus pulpous and endplate. The volume of the nucleus pulpous accounted for approximately 43% of the entire disc [13]. The thickness of each endplate was 0.4 mm, and zygapophysial cartilages were inserted into the space of the zygapophysial joints [13]. Eleven craniocervical and intervertebral ligaments were constructed based on their anatomical positions, including the cruciform ligament (transverse ligament (TL) and its vertical portion (CLV)), alar ligament (AL), apical ligament of the odontoid process (APL), anterior longitudinal ligament (ALL), posterior longitudinal ligament (PLL), ligamentum flavum (LF), capsular ligament (CL), interspinous ligament (ISL), supraspinous ligament (SSL) and intertransverse ligament (IL). According to the methods of Khuyagbaatar et al. [14], an FE model of the vertebral canal contents and nerve root complex (including the nerve roots, ventral and dorsal rootlets) was constructed (Fig. 1). In our model, we added the pia matter, which can play a role in the protection of white and grey matter [15]. The white and grey matter were constructed based on anatomical data from published anatomical texts and quantitative measurement studies [11, 12]. The pia matter (attached to the outer surface of white matter) and dura matter (located in the vertebral canal and placed 1.5–4.0 mm from the pia matter) were two layers of solid elements with thicknesses of 0.1 mm and 0.4 mm, respectively [15, 16]. The area between the pia matter and the dura matter was filled with CSF. DLs were constructed based on an available anatomical study; their wide base was attached to the lateral surface of the pia matter, and the apex was attached to the nerve roots at each spinal level [17]. Nerve roots forming from the ventral and dorsal nerve rootlets extended anterolaterally at an approximately 45° angle to the coronal plane [18]. Each nerve rootlet complex was assumed to have seven ventral and dorsal rootlets [19].

The whole FE model was constructed in the Cartesian coordinate system based on their anatomical positions (the *y*-axis is the sagittal direction of the model, while the *z*-axis is the axial direction). FE analysis software ABAQUS 2020 (Simulia/Dassault Systèmes, Vélizy-Villacoublay, France) was used to complete the pre-processing and analysis. The material properties were obtained from studies in the literature [20–22] (Table 1). DLs and nerve rootlets were modelled as 3D truss elements and were nonlinear, elastic–plastic and isotropic. In DL and nerve rootlet biomechanical characteristics studies, the yield point was obtained from their stress–strain curves [23, 24]. The pia matter and dura matter were elastic and isotropic [25–27], while the white and grey matter were nonlinear and hyperelastic [28]. The proximal side of each nerve root was tied on the dura matter, while the distal surface was coupled (kinematic method) with the adjacent vertebral body to ensure consistent movement between the two, as this closely simulates the condition of the human body. The proximal node of each DL was coupled (continuum distributing method) with the corresponding specific area of the pia matter to represent the wide base (Fig. 1). Smoothed particle hydrodynamics (SPH) analysis was used to simulate the interaction between CSF and the solid bodies. The CSF was converted to mass particles at the beginning of the analysis. SPH analysis can allow extreme deformation, and a dynamic, explicit analysis is required. Frictionless general contact was used in our model. Furthermore, convergence analysis was performed to ensure that the mesh density was acceptable. The whole FE model consisted of 957,789 nodes and 971,371 elements (Fig. 1).

**Table 1:** Material properties and element types used in the current model

Component	Element type	Material type	Material parameters	Cross-sectional area (mm^2^)	References
Cortical bone	Solid, C3D8R^a^	Elastic	*E*^c^ = 12,000 MPa	–	Huang et al. [20]
			*υ*^d^ = 0.29		
Cancellous bone	Solid, C3D8R	Elastic	*E* = 450 MPa	–	Huang et al. [20]
			*υ* = 0.29		
Zygapophysial cartilage	Solid, C3D8R	Elastic	*E* = 10 MPa	–	Huang et al. [20]
			*υ* = 0.3		
Annulus fibrosus	Solid, C3D8R	Elastic	*E* = 450 MPa	–	Huang et al. [20]
			*υ* = 0.4		
Nucleus pulpous	Solid, C3D8R	Elastic	*E* = 1 MPa	–	Huang et al. [20]
			*υ* = 0.49		
Endplate	Solid, C3D8R	Elastic	*E* = 1200 MPa	–	Huang et al. [20]
			*υ* = 0.29		
Grey matter	Solid, C3D8R	Hyperelastic (Ogden)	*μ*^e^ = 4.1 kPa, α^f^ = 14.7	–	Ichihara et al. [28]
White matter	Solid, C3D8R	Hyperelastic (Ogden)	*μ* = 4.0 kPa, α = 12.5	–	Ichihara et al. [28]
Pia matter	Solid, C3D8R	Elastic	*E* = 39.3 MPa	–	Jannesar et al. [25]
			*υ* = 0.3		
Dura matter	Solid, C3D8R	Elastic	*E* = 80 MPa	–	Persson et al. [27]
			*υ* = 0.49		
DLs	3D truss, T3D2^b^	Elasticplastic	Stress–strain curve	0.01	Polak et al. [23]
Nerve rootlet	3D truss, T3D2	Elasticplastic	Stress–strain curve	0.03	Singh et al. [24]
Nerve root	Solid, C3D8R	Elastic	*E* = 1.3 MPa	–	Nishida et al. [21]
			*υ* = 0.3		
CSF	Solid	Mie–Grüneisen equations of state, Newtonian fluid	co^g^ = 1,381,700 mm/s	–	Jannesar et al. [25] Panzer et al. [22]
			*s*^h^ = 1.979		
			Γ0^i^ = 0.11		
			*μ*^j^ = 0.0008 Pa s		

DLs, denticulate ligaments; CSF, cerebrospinal fluid

^a^An 8-node linear brick, reduced integration, hourglass control

^b^A 2-node linear 3D truss

^c^Elasticity modulus

^d^Poisson's ratio

^e^Shear modulus

^f^Strain hardening index

^g^Sound velocity

^h^A constant define the linear relationship between the shock velocity and the particle velocity

^i^Mie–Grüneisen ratio (A material constant)

^j^Viscosity

### Model validation

To comprehensively validate our FE model, ABAQUS 2020 was used to perform two conditions based on published in vivo and in vitro studies. First, a rotation of 20°/ − 20° around the *x*-axis was applied to simulate flexion and extension. The anterior/posterior (A/P), superior/inferior (S/I) and right/left (R/L) relative displacements of the C3–C7 spinal cord in the canal were calculated and compared to the in vivo data from magnetic resonance imaging (MRI) performed by Stoner et al. [29]. Second, we simulated the vertical compression of the cord according to a published in vitro study [30]. Specifically, a concentrated force of 0.8 N was applied to the middle segment of the spinal cord (perpendicular to the axial direction of the spinal cord) in the load module in ABAQUS 2020 to simulate vertical compression. The force–deformation curve was calculated and compared to the published in vitro study by Hung et al. [30]. In general, the data obtained from the validation were acceptable if they were within the mean value ± standard deviation ($\overline{x}$ ± SD) of the corresponding studies.

### CRM simulation

CRM, one of the most popular high-velocity and low-amplitude CSM techniques in China, was developed by Feng in the 1970s [6]. Three different positions (flexion, extension and neutral positions) of CRM were simulated in our study. Considering that most Chinese doctors are right-handed, only the right rotation position of CRM was studied. According to the principles of CRM recorded in Feng’s book [6], a Chinese manipulation textbook [31], our previous research [32] and published FE studies [33, 34], CRM loading was performed in the following steps. First, 15°, − 15° and 0° rotations around the *x*-axis were applied on the basilaris cranii to simulate the flexion, extension and neutral positions, respectively, and the basilaris cranii was axially rotated − 60° around *z*-axis (the right position) to its passive end range of motion [35]. Clinically, an upward traction force in the *z*-axis direction is applied to the basilaris cranii to counteract the gravity of the head at this time. Because we did not take the gravity of the head into consideration, the upward traction force was not applied in our simulation. Next, to enter the paraphysiological movement zone [35], a continuous axial rotation of 4° around the *z*-axis was applied to simulate high-velocity and low-amplitude CRM.

### Data analysis

The stress and strain of the spinal cord can be seen directly in the post-processing module of ABAQUS. In addition, the sagittal diameter, cross-sectional area of the spinal cord and distance from the spinal cord to the A/P wall of the vertebral canal were measured before and after CRM in the flexion, extension and neutral positions, respectively. Six equally divided levels were selected for each segment (C1–C7) to measure the data mentioned above. SPSS 21.0 statistical software (IBM Corporation, Armonk, New York, USA) was used for the data analysis. The measured data were expressed in the form of $\overline{x}$ ± SD. Statistically, because the data did not meet the assumptions of normality according to the Shapiro–Wilk test, significant differences among the data for CRM in the flexion, extension and neutral position were determined by the Mann-Whiney test. The Wilcoxon signed-rank test was used to identify differences between the data before CRM and the data after CRM for each position. Significant differences were defined with *P* values < 0.05.

## Results

### Validation of the FE model

**Fig. 2:**
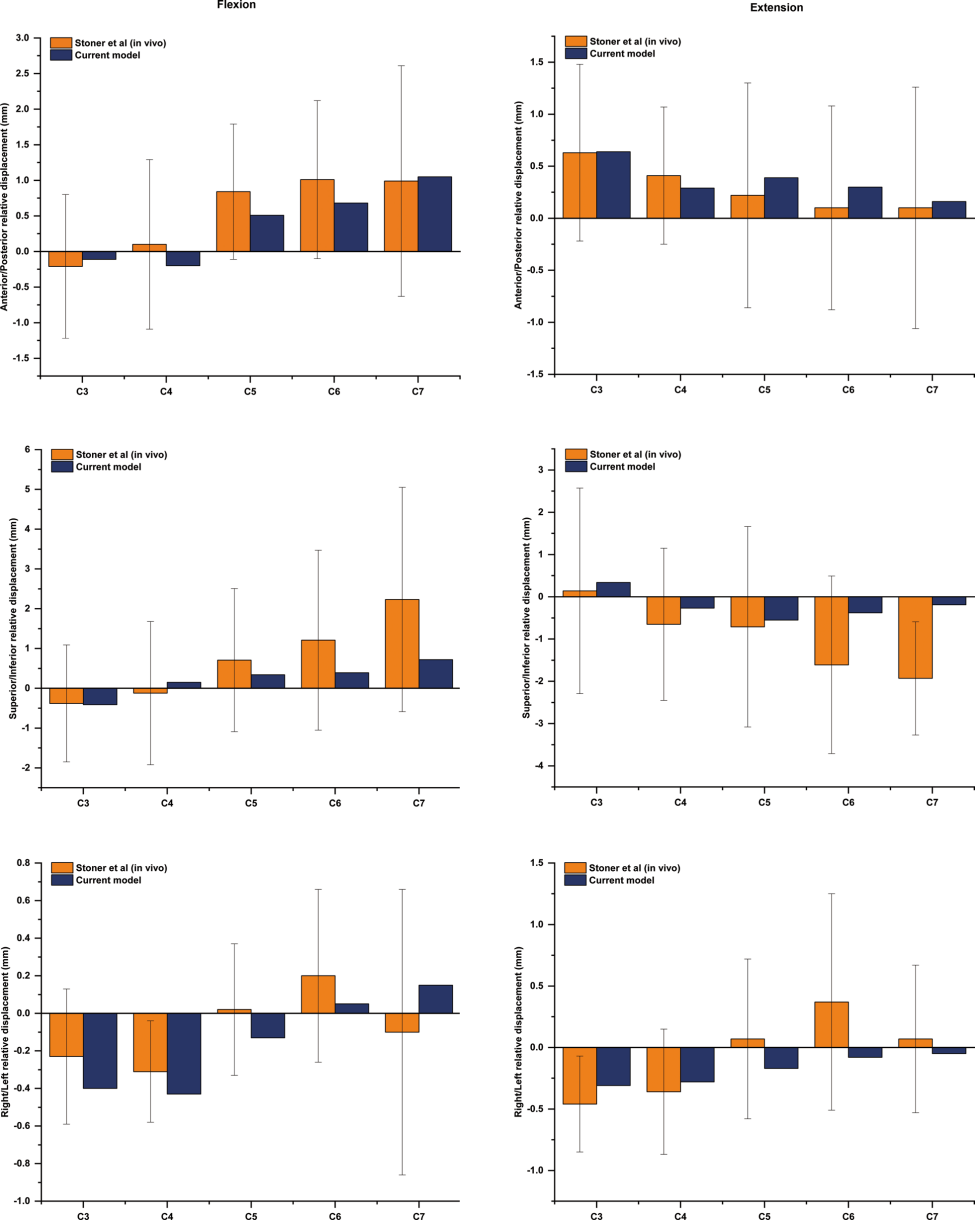
Displacement of the cervical spinal cord in the flexion and extension positions and comparison to the in vivo study by Stoner et al. The positive values indicate displacement in the anterior, superior or right direction. (A/P, anterior/posterior; S/I, superior/inferior; R/L, right/left)

**Fig. 3:**
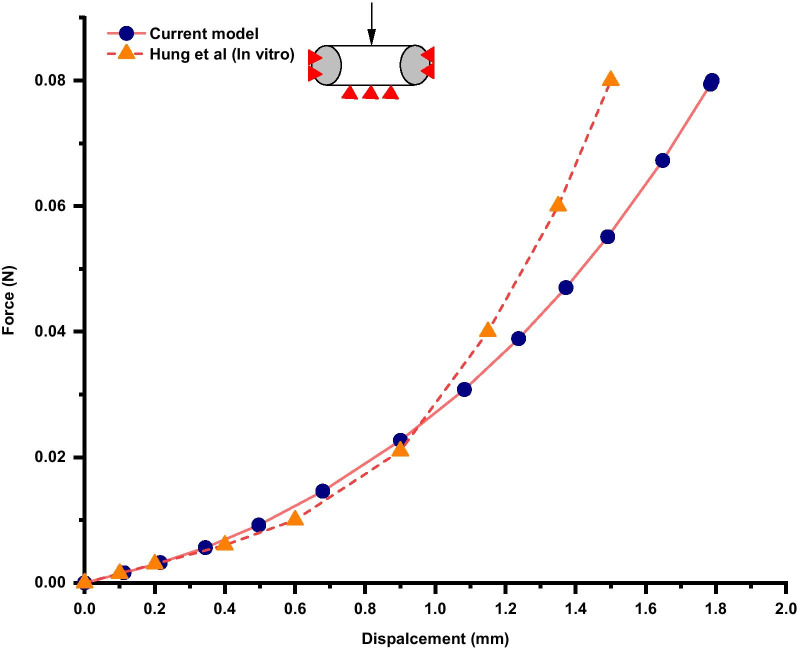
Force–deformation curve of the spinal cord vertical compression test and comparison to the in vitro study by Hung et al.

We validated our FE model against the published data mentioned previously. To determine whether the whole spinal cord, including the white matter, grey matter, nerve rootlets, CSF, etc., was acceptable, we calculated the A/P, S/I and R/L displacement of the spinal cord in the C3–C7 segments, and the results were adequately close to the MRI measurements [29] (Fig. 2). We could see a turning point at the C3/4 segment in flexion, while the C3 segment of the spinal cord moved inferiorly and the C7 segment of the spinal cord moved superiorly. The opposite occurred in extension, where the C3 segment of the spinal cord moved superiorly and other segments of the cord moved inferiorly. The values of the S/I displacement in the C4–C7 segments were smaller than those in the MRI measurements, but they were within the mean value ± standard deviation. The force–deformation curve of the spinal cord vertical compression test is shown in Fig. 3, which matched the results of the in vitro study well [30]. A nearly linear relationship in the curve is seen when the deformation is lower than 0.4 mm, while a nonlinear relationship appears when the load is increased. The findings indicate that the spinal cord kinematic characteristics of the current model can represent a statistically healthy individual.

### Stress distribution on the spinal cord

**Fig. 4:**
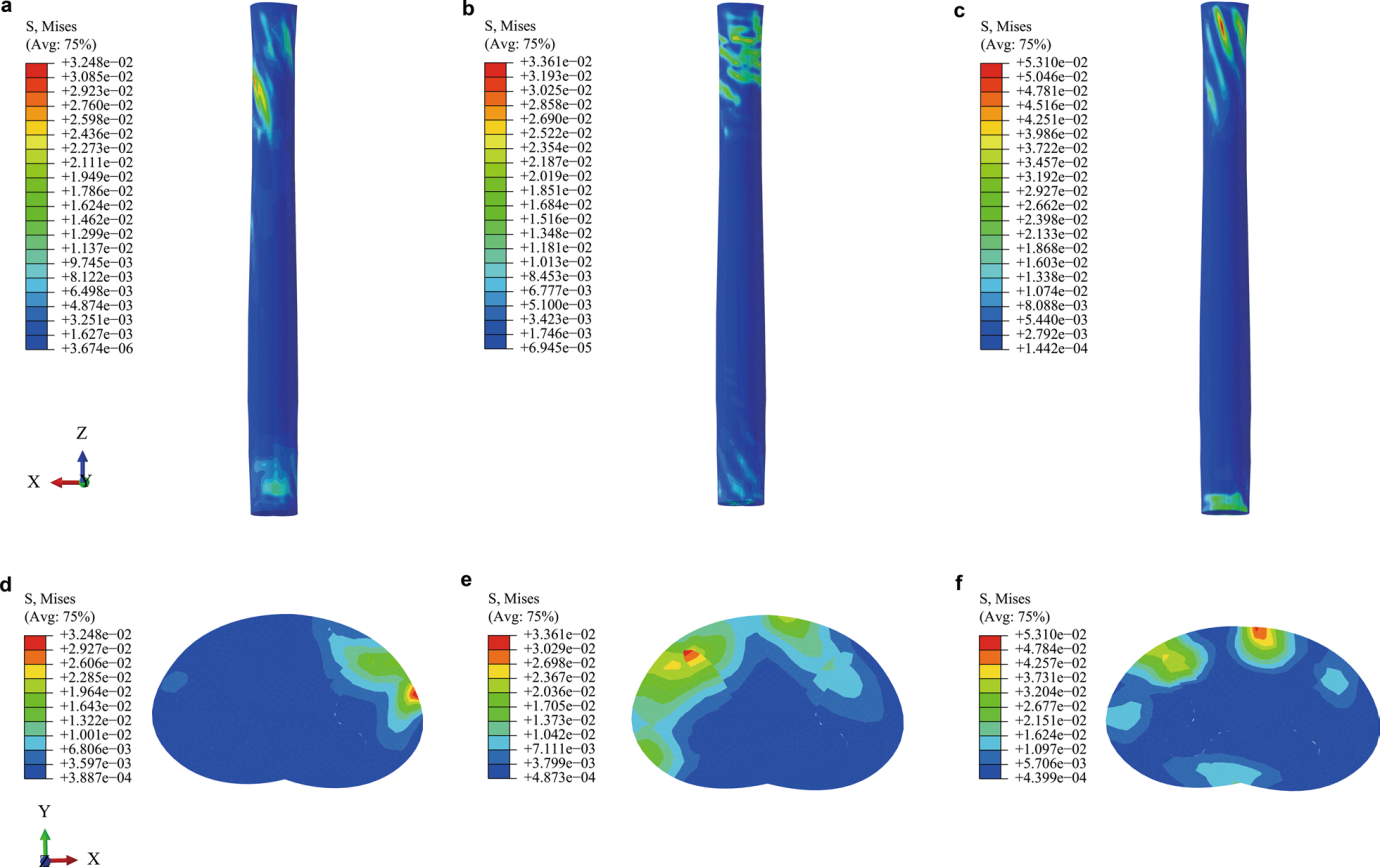
Stress distribution on the spinal cord. **a** Stress distribution on the spinal cord after CRM in the flexion position.** b** Stress distribution on the spinal cord after CRM in the extension position.** c** Stress distribution on the spinal cord after CRM in the neutral position. **d** Cross-sectional stress distribution on the segment with the maximum stress after CRM in the flexion position. **e** Cross-sectional stress distribution on the segment with the maximum stress after CRM in the extension position.** f** Cross-sectional stress distribution on the segment with the maximum stress after CRM in the neutral position. (CRM, cervical rotatory manipulation)

Before the high-velocity and low-amplitude 4° *z*-axis rotation during CRM, the maximum von Mises stress on the spinal cord was roughly equal in the flexion, extension and neutral positions (0.0211, 0.0212, 0.0200 MPa, respectively). After the rightward 4° *z*-axis rotation during CRM, the maximum von Mises stress on the spinal cord in the extension and neutral positions was higher than that in the flexion position (Fig. 4). The maximum von Mises stress on the spinal cord after CRM was at the C2, C1 and C1 segments in the flexion, extension and neutral positions, respectively. Compared with the value before the 4° *z*-axis rotation, the maximum von Mises stress increased by 53.6%, 58.5% and 164.97% after CRM in the flexion, extension and neutral positions, respectively. Additionally, higher von Mises stress was seen on the left-posterior side of the spinal cord after CRM in the flexion position, while higher von Mises stress was seen on the right-posterior side of the spinal cord after CRM in the extension position (Fig. 4). The maximum von Mises stress in the flexion and neutral positions was almost located on the white matter, while the maximum von Mises stress after CRM in the extension position was almost located on the grey matter (Fig. 4). The strain distribution on the spinal cord followed the stress distribution.

### Sagittal diameter and cross-sectional area of the spinal cord

Before and after CRM, we measured the sagittal diameter and the cross-sectional area of the spinal cord to evaluate its free space in the vertebral canal.

**Fig. 5:**
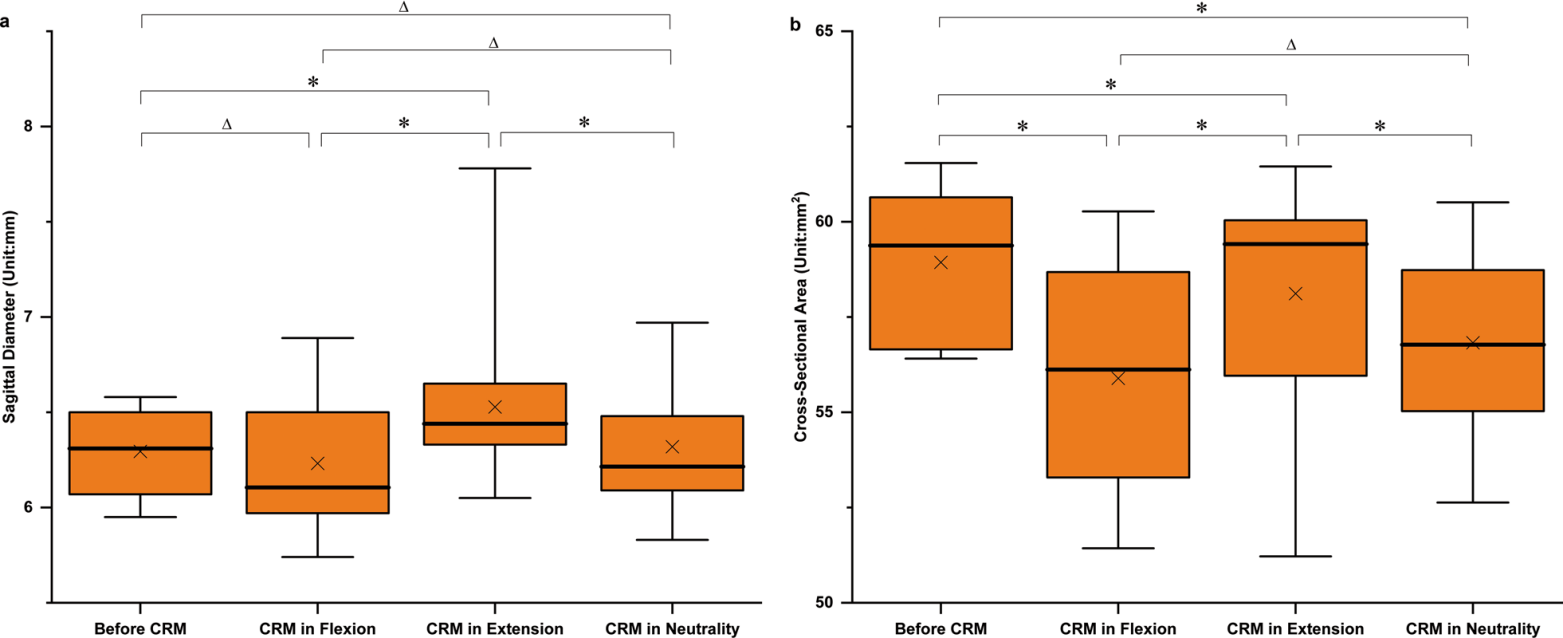
Boxplots of the sagittal diameter and cross-sectional area of the spinal cord. On each box, the central thick line mark indicates the medians, the “x’’ marker indicates the means, and the top and bottom edges indicate the 75th and 25th percentiles, respectively. The whiskers extend to the maximum and minimum values not considered outliers. *P < .05. Δ *P* > .05 (CRM, cervical rotatory manipulation)

The average sagittal diameters of the spinal cord were 6.23 mm, 6.53 mm and 6.32 mm after CRM in the flexion, extension and neutral positions, respectively. In general, compared with the value before CRM, the sagittal diameter of the spinal cord after CRM in the extension position was increased (*P* < 0.001). However, there was no significant difference between the sagittal diameters of the spinal cord before and after CRM in the flexion and neutral positions (*P* = 0.096, *P* = 0.630, respectively) (Fig. 5). Specifically, compared with the value in the extension and neutral positions, the sagittal diameter of the spinal cord decreased by 4.6% (*P* < 0.001) and 1.4% (*P* = 0.105) in the flexion position, respectively. Compared with the value in the flexion and neutral positions, the sagittal diameter of the spinal cord increased by 4.8% (*P* < 0.001) and 3.3% (*P* = 0.002) in the extension position, respectively. Furthermore, the minimum and maximum sagittal diameters of the spinal were found at the C6 segment after CRM in the flexion position and at the C1 segment after CRM in the extension position, respectively (Table 2).

**Table 2:** Sagittal diameters of the spinal cord (unit: mm)

	C1 (*n* = 6)	C2 (*n* = 6)	C3 (*n* = 6)	C4 (*n* = 6)	C5 (*n* = 6)	C6 (*n* = 6)	C7 (*n* = 6)
Before CRM	6.50 ± 0.03	6.54 ± 0.04	6.47 ± 0.05	6.26 ± 0.05	6.03 ± 0.04	6.03 ± 0.03	6.24 ± 0.11
CRM in flexion	6.65 ± 0.18	6.60 ± 0.21	6.09 ± 0.13	6.36 ± 0.16	5.96 ± 0.07	5.95 ± 0.05	6.01 ± 0.14
CRM in extension	7.16 ± 0.38	6.63 ± 0.21	6.62 ± 0.05	6.46 ± 0.12	6.20 ± 0.12	6.27 ± 0.11	6.36 ± 0.05
CRM in neutrality	6.78 ± 0.09	6.65 ± 0.25	6.23 ± 0.04	6.38 ± 0.09	6.10 ± 0.1	6.00 ± 0.1	6.10 ± 0.06

Data expressed as *x̅* ± SD

CRM, cervical rotatory manipulation

Interestingly, the cross-sectional area of the spinal cord decreased after CRM in the flexion, extension and neutral positions compared with the value before CRM (*P* ≤ 0.001). Specifically, the cross-sectional area of the spinal cord after CRM in the extension position was 4.0% (*P* < 0.001) and 2.1% (*P* = 0.007) higher than the value in the flexion and neutral positions, respectively (Fig. 5). An upward trend in the cross-sectional areas from C2 to C5 after CRM in the three different positions was observed, with maximum values at C5 and C6. The maximum and minimum cross-sectional areas were found at segment C5 after CRM in the extension position and at segment C2 after CRM in the flexion position, respectively (Table 3).

**Table 3:** Cross-sectional areas of the spinal cord (unit: mm^2^)

	C1 (*n* = 6)	C2 (*n* = 6)	C3 (*n* = 6)	C4 (*n* = 6)	C5 (*n* = 6)	C6 (*n* = 6)	C7 (*n* = 6)
Before CRM	57.73 ± 1.80	56.47 ± 0.04	57.45 ± 0.64	59.58 ± 0.87	61.04 ± 0.24	60.98 ± 0.42	59.30 ± 0.67
CRM in flexion	54.73 ± 2.45	52.71 ± 0.85	52.82 ± 1.06	56.02 ± 0.90	58.81 ± 0.54	59.49 ± 0.47	56.68 ± 0.95
CRM in extension	53.82 ± 2.31	55.77 ± 1.23	56.88 ± 1.22	60.03 ± 0.50	60.55 ± 0.40	60.34 ± 0.74	59.40 ± 0.48
CRM in neutrality	53.58 ± 1.10	55.02 ± 1.25	55.32 ± 0.67	57.27 ± 0.71	59.7 ± 0.55	59.41 ± 0.74	57.45 ± 0.86

Data expressed as *x̅* ± SD

CRM, cervical rotatory manipulation

### Anterior/posterior eccentricity of the spinal cord

Before and after CRM, the distance from the spinal cord to the A/P wall of the vertebral canal was measured, and the A/P eccentricity of the spinal cord was calculated to represent the A/P position of the cord because the compression of the spinal cord by structures often originates from these two directions.

**Fig. 6:**
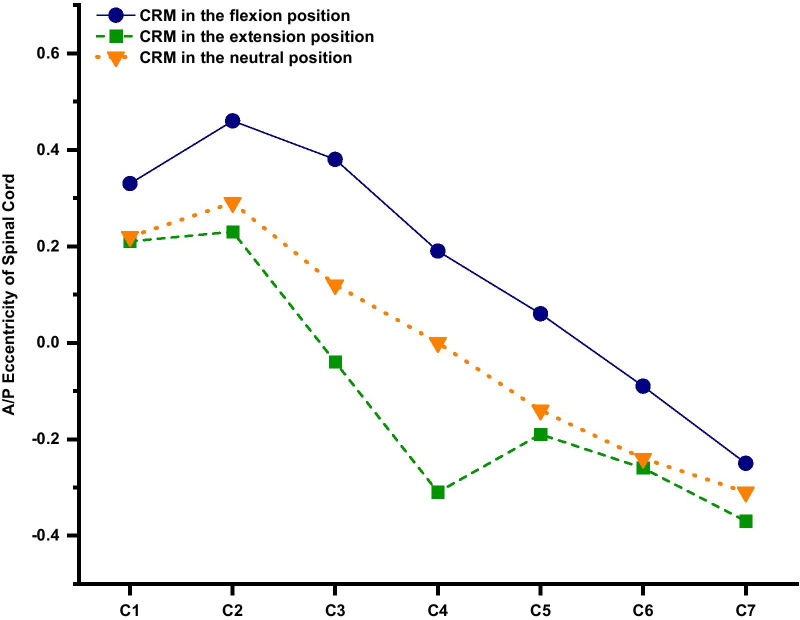
A/P eccentricity of the cervical spinal cord. ${\text{A/P}}\;{\text{eccentricity}} = \left( {\frac{P - A}{{P + A}}} \right)$, where *P* is the distance from the spinal cord to the posterior wall of the vertebral canal, and *A* is the distance from the spinal cord to the anterior wall of the vertebral canal. An *A*/*P* eccentricity > 0 (< 0) indicates that the spinal cord is anteriorly (posteriorly) positioned (CRM, cervical rotatory manipulation; A, anterior; P, posterior)

A/P eccentricity follows the formula:

$${\text{A/P}}\;{\text{eccentricity}} = \left( {\frac{P - A}{{P + A}}} \right)$$

where *P* is the distance from the spinal cord to the posterior wall of the vertebral canal and *A* is the distance from the spinal cord to the anterior wall of the vertebral canal. An A/P eccentricity > 0 (< 0) indicates that the spinal cord is anteriorly (posteriorly) positioned. The spinal cord is anteriorly positioned after CRM in the flexion position, while the spinal cord is posteriorly positioned after CRM in the extension and neutral positions (Fig. 6).

## Discussion

Our study aimed to explore the biomechanical and kinematic characteristics of the spinal cord in CRM in the flexion, extension and neutral positions and the relationship between the characteristics and clinical applications of CRM. We constructed and validated a 3D craniocervical FE model of the spinal cord and nerve root complex. During our validation, smaller S/I displacement was seen at the C4–C7 segments compared to MRI in vivo measurements by Stoner et al. [29]. We speculate that the reason for this phenomenon is that the present study constructed the cervical spinal cord, but the load effects on the thoracic, lumbar spinal cord and brain were not taken into account. Nevertheless, the S/I displacement value was within the mean value ± standard deviation.

In this study, we found that the maximum von Mises stress occurred at the C1 or C2 segment after CRM in the three different positions, which is closely related to the ranges of axial rotation at the atlantoaxial joint. This joint articulates with three synovial joints: two lateral joints and a single median joint, and allows 50% of the axial rotation at the cervical spine [36]. According to Panjabi et al., the range of unilateral axial rotation at the atlantoaxial joint is 28°–40° [37]. Consequently, the C1–C2 segments of the spinal cord experienced higher stress when high-velocity and low-amplitude 4° *z*-axis rotation was performed, which demonstrates that the C1–C2 segments of the spinal cord were more likely to be injured.

Additionally, CRM in different positions led to different stress and strain on the spinal cord. The spinal cord experienced higher von Mises stress in CRM in the extension and neutral positions, which demonstrated that, under the same degrees of axial rotation, CRM in the flexion position may be less likely to cause spinal cord injury. Furthermore, from the cross-sectional stress distribution on the segment with maximum stress in Fig. 4, we can see that the maximum von Mises stress after CRM in the extension position was located on the grey matter. Ichihara et al. reported that the grey matter ruptured at lower strains than the white matter [28]. Accordingly, the higher von Mises stress on the grey matter after CRM in the extension position was more likely to cause spinal cord injury.

To evaluate the free space of the cervical spinal cord in the vertebral canal, the sagittal diameter and the cross-sectional area of the spinal cord were measured. Lower sagittal diameter and cross-sectional area can be seen after CRM in the flexion and neutral positions, which demonstrated that the spinal cord after CRM in the flexion and neutral positions has more free space to buffer the influence of other structures. This finding is in line with the study of continuous measurements of the cervical spinal cord in single flexion based on MRI that the ratio of the sagittal diameter to the transverse diameter of the spinal cord is lower in the flexion position, and similar to the cross-sectional area of the spinal cord [38]. However, because of cervical enlargement, the maximum cross-sectional area of the spinal cord was located at segments C5 and C6 before and after CRM in the three different positions. Although the overall cross-sectional area after CRM in the flexion or neutral position was lower, the cross-sectional area in the C6 segment was roughly equal after CRM in the three positions. That is, the free space of the spinal cord at the C6 segment after CRM in the flexion or neutral position was also relatively narrow. The spinal cord was likely to experience compression injury in this segment. In addition, compared with the cross-sectional area of the spinal cord before CRM, the value after CRM was significantly lower, which may demonstrate that CRM in all three positions is safe for a healthy individual in terms of the cross-sectional area.

Regarding A/P eccentricity, we found that the spinal cord followed the motion of the vertebral canal, with more free space posteriorly after CRM in the flexion position than in the extension and neutral positions, which is in line with the literature [39]. There is a change in the length of the vertebral canal when the cervical spine moves in flexion or extension, and the dura and cord stretch or compress to compensate for this change. Accordingly, when flexion occurs, the stretch tension in the kyphotic dura and cord will generate a forward force that causes the dura to approach the anterior wall of the vertebral canal, and the spinal cord is pulled by the DLs and moves in parallel with the dura [40]. Thus, posterior vertebral osteophytes or disc herniations are likely to influence the cord after CRM in the flexion position.

Clinically, we should obtain a detailed medical history and perform a physical examination and primary radiographic evaluation before CRM [41, 42]. It was reported that cervical intradural disc herniation may cause progressive neurological dysfunction after cervical spine manipulation therapy [43]. Similarly, in this study, we found that CRM may not be suitable if disc herniation or a vertebral osteophyte is present around the C1–C2 segments because the maximum stress of the spinal cord occurs at these segments after CRM, and disc herniation or a vertebral osteophyte may cause much higher stress, thereby leading to spinal cord injury. Furthermore, the results showed that CRM performed in the flexion position may be a better choice because the procedure in this position is associated with lower von Mises stress on the spinal cord and sufficient free space in the vertebral canal [39], which is in line with Feng’s theory [6]. However, CRM in the flexion position is not suitable if there is a large posterior vertebral osteophyte or disc herniation, especially in the C6 segment (a segment with a high prevalence of such diseases) [41, 44], because the spinal cord is anteriorly positioned after CRM in the flexion position. If CRM in the extension position has to be performed, a large ossification or thickening of the LF should be excluded in advance [45].

We constructed a detailed FE model with the aim of obtaining accurate results, but there are also some limitations in this study. First, the loading and boundary conditions were simplified (i.e., traction force was not applied, because it exactly counteracted the gravity of the head), and they were based on a Chinese manipulation textbook [6, 31], our previous research [32] and published finite element studies [33, 34], which may not precisely replicate the clinical situation but is helpful in terms of comparability and reproducibility. Second, the study is based on an FE model that was constructed based on data from only one healthy young male. The material properties of females and elderly individuals may be different, and more models with different ages and sexes are needed in future studies. Third, this study did not quantitatively elucidate the influence of disc herniation, vertebral osteophytes or LF ossification on the spinal cord. However, this influence is unlikely to alter the findings of this study.

## Conclusions

This simulation model of CRM in the flexion, extension and neutral positions can be used to predict the biomechanical and kinematic characteristics of the spinal cord and to help guide clinical applications of CRM. The results suggested that CRM could significantly alter the stress distribution, the forma and the relative position of the spinal cord. Clinically, CRM should be used with caution if there is a disease such as disc herniation or a vertebral osteophyte around the C1–C2 segments because the results showed that maximum von Mises stress of the spinal cord occurs at these segments after CRM. In addition, we found that CRM in the flexion position has lower von Mises stress on the spinal cord and sufficient free space in the vertebral canal. Thus, CRM in the flexion position could be a better choice than CRM in the extension and neutral positions, but caution is warranted if posterior vertebral osteophytes or disc herniations are present, especially in the C6 segment.

## Data Availability

The data used and/or analysed during the current study are available from the corresponding author or the first author on reasonable request.

## Abbreviations

CSM: Cervical spine manipulation
CRM: Cervical rotatory manipulation
FE: Finite element
CSF: Cerebrospinal fluid
DLs: Denticulate ligaments
CT: Computed tomography
